# Interfacial Route to Low-Fat Muffin Cake Quality: Pre-Emulsification-Enabled Lipase Action Improves Structure and Acceptance

**DOI:** 10.3390/foods15060978

**Published:** 2026-03-10

**Authors:** Simge Ozbek, Emrah Kirtil

**Affiliations:** 1 Pak Gıda Üretim ve Pazarlama A.Ş., 41250 Kocaeli, Türkiye; s.ozbek2024@gtu.edu.tr; 2 Department of Chemical Engineering, Gebze Technical University, 41400 Kocaeli, Türkiye

**Keywords:** low-fat cake, lipase, pre-emulsification, crumb texture, sensory analysis, clean label

## Abstract

Reducing cake fat while maintaining aeration, crumb softness, and consumer acceptance remains challenging because fat crystals contribute to interfacial stabilization and structure development. This study evaluated an interfacial processing strategy in which oil dispersion is refined by pre-emulsification to evaluate whether refining oil dispersion by pre-emulsification modulates the functional impact of lipase (via in situ formation of surface-active lipolysis products). A D-optimal design (16 formulations) quantified the effects of fat type (shortening vs. sunflower oil), fat level (100% vs. 50%), pre-emulsification (absent/present), and lipase dose (0, 50, 100 ppm; flour basis) on batter and baked-cake quality. Responses included moisture, color, volume/visual structure, texture and hedonic sensory evaluation for selected formulations. Lipase improved structure and texture, with the strongest benefits in reduced-fat samples, where hardness-related parameters decreased and volume/crumb refinement improved. Pre-emulsification modulated lipase performance in a formulation-dependent manner, indicating significant interactions. In sensory tests, the combined approach improved low-fat acceptance compared with the low-fat control. Overall, pre-emulsification-enabled lipase action offers a route to recover key quality attributes in low-fat cakes without conventional emulsifiers.

## 1. Introduction

Dietary guidance and regulatory pressure continue to push the bakery sector toward reducing saturated-fat intake and eliminating industrial trans fats (typically associated with partially hydrogenated fats), motivating reformulation of fat-rich products such as cakes while preserving consumer-expected quality attributes [1]. Yet, in aerated cakes, fat is not merely a caloric contributor; it is a primary structuring agent that governs batter aeration, bubble stability, crumb softness, and the kinetics of firming during storage [2]. In particular, crystalline shortening supports the formation and stabilization of finely dispersed air cells during mixing, which is essential for achieving high specific volume and a uniform crumb [3,4]. Consequently, fat reduction or direct substitution of solid fat with liquid oils often produces denser batters, coarser bubble populations, lower volume, and accelerated textural deterioration which are outcomes that limit practical adoption even when the nutritional intent is clear [2,5].

Mechanistically, cake batter can be viewed as a multiphase system in which aeration and baking expansion depend on the coupled stability of air–water and oil–water inter-faces. Brooker’s classic microstructural model describes how fat crystals, once coated by interfacially adsorbed proteins, can attach to air bubble surfaces and provide an interfacial “reserve” that assists bubble expansion during heating—an explanation for why oils generally do not replicate the aeration performance of crystalline fats at equal mass [3]. This framework clarifies the central bottleneck in “health-driven” reformulation: reducing solid fat content removes a key interfacial stabilizer precisely at the stage when the batter must retain many small bubbles without coalescence or collapse [2]. Ingredient and process interventions must therefore (i) restore interfacial stability, (ii) maintain an appropriate rheological window for air incorporation and retention, and (iii) do so without introducing unacceptable additives or sacrificing shelf-life [2].

Accordingly, the literature can be organized into a few dominant solution families. First, bulk fat replacers (e.g., inulin, modified starches, pregelatinized/extruded flours, protein or hydrocolloid systems) aim to rebuild viscosity and moisture retention, often improving tenderness but not always recovering aeration and grain uniformity [2,5]. Second, emulsifier-based strategies attempt to compensate for the loss of solid fat by accelerating adsorption at interfaces and strengthening films around bubbles and droplets; these approaches can mitigate hardness and volume losses, but they may be constrained by labeling preferences and formulation-dependent trade-offs [6]. Third, emerging “structuring” routes (e.g., engineered liquid shortenings or oleogel-like concepts) seek to mimic shortening functionality; however, implementation complexity and ingredient constraints remain nontrivial in conventional cake processes [4].

A design logic that follows from these constraints is to restore interfacial functionality by generating surface-active species in situ, thereby stabilizing both oil droplets and gas cells even when solid fat is reduced and/or replaced with liquid oil. Enzymatic lipid modification via lipases directly targets this bottleneck: by hydrolyzing triglycerides, lipases produce mono- and diacylglycerols and free fatty acids that act as powerful amphiphiles, functionally resembling conventional emulsifiers while remaining compatible with clean-label positioning [7,8]. In this framework, pre-emulsification is a supportive processing aid that can increase accessible interfacial area by refining oil dispersion before mixing, potentially facilitating lipase action and improving aeration stability [2].

Evidence supporting this enzymatic route has begun to accumulate in cake systems. Lipase addition has been reported to improve cake quality attributes in practical manufacture settings, consistent with enhanced interfacial functionality and batter structuring [8,9,10,11]. In reduced-fat formulations, combining lipase with formulation adjustments has also been explored as a means to recover textural and structural performance when fat is partially replaced (e.g., by inulin), reinforcing the notion that lipase-generated amphiphiles can contribute to crumb refinement and softening under constrained fat conditions [12]. More recently, controlled comparisons have shown that selected lipases can improve cake baking properties relative to a traditional emulsifier benchmark, underscoring their potential as targeted functional improvers rather than merely “processing enzymes” [8].

In this context, a central hypothesis emerges: pre-emulsification can increase accessible interfacial area and improve lipid dispersion, thereby enabling lipase activity to generate sufficient in situ surface-active species to stabilize batter foams and emulsions, ultimately preserving cake volume and crumb softness despite (i) a shift from crystalline shortening to liquid oil and/or (ii) a meaningful reduction in total fat. This hypothesis is inherently testable through coupled measurements of batter behavior (e.g., density/specific gravity proxies, stability) and baked quality endpoints (e.g., specific volume and texture evolution), while explicitly resolving interactions among fat type, fat level, and process choices.

To test this design proposition efficiently, the present study applies a D-optimal experimental design (a standard approach for extracting main and interaction effects with fewer runs than full factorial experimentation) [13] to select 16 formulations spanning four factors: fat type (shortening vs. sunflower oil), fat level (100% vs. 50%), presence/absence of a pre-emulsification step, and lipase dosage (0/50/100 ppm, flour basis). Pre-emulsification is implemented by high-shear homogenization of the full fat phase with 20% of the formulation water for 60 s prior to batter mixing, and batter resting time is fixed at 30 min to control process history. Overall, the objective is to quantify how enzymatic lipid modification and pre-emulsification—individually and synergistically—can offset the functional losses associated with fat reduction and oil substitution, and to identify formulation–process combinations that best maintain cake structural and textural performance.

## 2. Materials and Methods

### 2.1. Materials

All-purpose flour (Söke, Turkey), sugar (Torku, Turkey), egg powder (Pormova, Turkey), salt (Billur Tuz, Turkey), and baking powder (Pakmaya, Turkey) were purchased from a local grocery store (Migros, Turkey). The fat phase was provided either as sunflower oil (Komili, Turkey) or as vegetable shortening, in accordance with the experimental design (Table 1). The vegetable shortening (Ustam, Marsa Yağ San. ve Tic. A.Ş., Turkey) is an anhydrous, trans-fat-free bakery fat composed of a vegetable-oil/fat blend (palm, cottonseed, canola, and sunflower) in varying proportions as declared by the manufacturer (exact percentages not specified). A commercial food-grade baking lipase preparation (Lipopan^®^ Xtra, Novonesis/Novozymes A/S, Bagsværd, Denmark) was used as the enzymatic improver and dosed on a flour-weight basis as specified in the experimental design. Consumables for batter handling and baking included disposable piping bags, paper muffin cups, and standard laboratory items for weighing, labeling, and sample storage. For sensory sessions, drinking water and plain crackers (Cubuk Kraker, Ulker, Turkey) were provided as palate cleansers. All ingredients were stored under controlled temperature and humidity to ensure product consistency and integrity prior to use.

### 2.2. Methods

#### 2.2.1. Experimental Design

A D-optimal experimental design was implemented to directly address the study objective: to quantify how fat type (solid shortening, S; liquid sunflower oil, L), fat reduction level (100% vs. 50%), lipase dose (Lip0/Lip50/Lip100 at 0/50/100 ppm on a flour-weight basis; ppm = mg enzyme preparation per kg flour), and lipase delivery strategy via pre-emulsification (P0: no; P1: yes) influence cake performance, including potential factor interactions (e.g., whether lipase efficacy depends on fat type and/or fat reduction). The candidate formulation space followed a 2 × 2 × 2 × 3 factorial structure, while resting time before baking was fixed at 30 min to remove an additional processing source of variability. A D-optimal subset of 16 formulations (sample codes listed in Table 1) was selected to obtain an information-efficient set of runs capable of estimating the targeted effects under practical constraints [14].

#### 2.2.2. Cake Batter Preparation and Baking

Cake batters were prepared using a constant base formulation (expressed on a flour basis): for every 100 g flour, sugar (100 g), fat (25 g for the control-fat level; 12.5 g for the 50% fat-reduced level), egg powder (9 g; used in place of fresh eggs), salt (2 g), baking powder (4 g), and water (77.4 g). The fixed water addition corresponds to 36.0 g water per 100 g total dry ingredients (i.e., flour + sugar + egg powder + salt + baking powder). Only fat type (solid shortening vs. liquid sunflower oil), fat level (100% vs. 50%), pre-emulsification (P0 vs. P1), and lipase dose (0/50/100 ppm, flour basis) varied according to the experimental design (Table 1). Mixing and pre-emulsification (where applicable) were performed using a planetary mixer with a paddle attachment (N50, Hobart, Troy, OH, USA).

For non-pre-emulsified batters (P0), all ingredients were combined directly and mixed for 30 s at speed 1, followed by 3 min 30 s at speed 2. For pre-emulsified batters (P1), an oil-in-water pre-emulsion was first prepared by mixing the entire fat phase with 20% of the formulation water for 60 s at speed 3; the remaining 80% water, egg, and cake mix were then added and the batter was mixed for 30 s at speed 1 and 3 min 30 s at speed 2. The batter preparation was performed at room temperature.

Lipase was applied at 0, 50, or 100 ppm on a flour-weight basis (mg enzyme preparation per kg flour). In pre-emulsified treatments (P1), the enzyme dose (Lip50 or Lip100) was first dissolved in the 20% water fraction and incorporated during the pre-emulsification step together with the fat phase (60 s at speed 3), before the remaining ingredients were added. For shortening-based formulations, the shortening was gently melted to a fully liquid state immediately before this step so that it could be dispersed into the water fraction under the same high-shear conditions. In non-pre-emulsified treatments (P0), the lipase solution was added during the main mixing stage together with the other ingredients. In contrast, for P0 treatments, the lipase solution was incorporated during the main mixing stage together with the remaining water and ingredients. For Lip0 controls, the P1 workflow was performed with-out lipase to decouple the physical effect of pre-emulsification from enzymatic action, consistent with the interfacial nature of lipase activity and prior use of low-dose lipase as a clean-label improver in reduced-fat cake systems [15].

After mixing, batters were transferred to piping bags, sealed to minimize moisture loss, and rested for 30 min at ambient conditions. Batters were then portioned (80 g) into paper muffin cups and baked Unox Rossella convection oven (Model XF195-B; Unox S.p.A., Padua, Italy) under fan-assisted conditions at 165 ◦C for 23 min. Baked cakes were cooled at ambient temperature (22–25 °C) until reaching room temperature (typically ~45–60 min for the muffin-sized portions used). After cooling, each cake was covered with polyethylene wrapping film (plastic wrap) to minimize moisture loss. The wrapped cakes were then stored overnight at ambient temperature until the following day prior to analyses. Standardized control of processing (fixed mixing, resting, and baking conditions across treatments) follows common practice in cake-baking test methods intended to isolate formulation effects [16].

#### 2.2.3. Moisture Content Analysis

Moisture content of the baked cakes was determined the day after baking by a gravimetric loss-on-drying approach using a halogen moisture analyzer (HB43-S, Mettler Toledo, Greifensee, Switzerland). Briefly, approximately 3.4–4.6 g of cake crumb was weighed into the analyzer and dried under the instrument’s standard drying conditions until completion of the drying program; moisture (%) was calculated from the mass loss upon drying relative to the initial mass.

#### 2.2.4. Color Measurement

Cake color was measured using a benchtop colorimeter (CR-5, Konica Minolta, Tokyo, Japan) the day after baking, after cooling to ambient temperature and overnight holding. Prior to measurements, the instrument underwent user dark and white calibration with the manufacturer’s calibration accessories (zero calibration box and white calibration plate) to ensure operation within the defined reflectance range. Measurements were acquired in the CIE 1976 Lab* color space and reported as L* (lightness), a* (red–green axis), and b* (yellow–blue axis). In addition, overall color difference (∆E*) was calculated in the CIE 1976 Lab* framework as the Euclidean distance from the Lab* origin (*L*_0_ = 0, *a*_0_ = 0, b_0_ = 0) using the following equation;

E* = √(L*^2^ + a*^2^ + b*^2^).

Crust and crumb were evaluated separately. For crust color, six readings were taken per cake: one from the top central region, one from the bottom central region, and four readings from the lateral faces. These six readings were not retained as separate values; instead, their average was used as the single crust color value for that cake. For crumb color, cakes were cut at the midline and measurements were taken from the midpoint of each exposed cut surface (two readings per cake); the two readings were averaged to obtain a single crumb color value. ∆E* was computed separately for crust and crumb using the corresponding averaged L*, a*, and b* values for each cake, with the same reference definition applied consistently within each matrix (crust or crumb).

#### 2.2.5. Volume Measurement

Cake volume was determined the day after baking, after cooling and overnight holding, using a laser-based volume meter (TexVol BVM-L450LC, Perten Instruments, Hägersten, Sweden). The method is based on laser topography, in which the sample is placed on a rotating platform and a laser sensor scans the product surface along a defined trajectory to reconstruct a three-dimensional surface profile, from which volume (mL) is computed [17]. Each cake was mounted in the instrument’s measurement chamber as a whole (secured on the support shaft as specified for stable positioning during rotation), the scan was initiated via the instrument software, and the volume output (mL) reported by the system was recorded for subsequent analysis.

#### 2.2.6. Visual Analysis (Image-Based Evaluation)

Representative digital photographs were acquired for each formulation to document external appearance (crust) and internal structure (crumb) at the same post-bake timepoint as the instrumental analyses (i.e., after cooling and overnight holding). For crust imaging, each muffin cake was photographed intact in an upright position to capture overall shape (doming), surface uniformity, and visible defects (e.g., cracking). For crumb imaging, each cake was cut through the geometric midline, and the two halves were positioned with the fresh cut surfaces facing the camera to capture the central crumb region. To minimize perspective-related distortion and ensure between-sample comparability, photographs were taken using a fixed imaging geometry (constant camera-to-sample distance and alignment with the sample midline) and a consistent, neutral background and placement surface; images were labeled using the corresponding sample codes (Table 1).

#### 2.2.7. Texture Profile Analysis

Instrumental texture was evaluated using a texture analyzer (CT3 Texture Analyzer, AMETEK Brookfield, Middleboro, MA, USA) equipped with a cylindrical compression probe (TA-AACC36; 36 mm diameter). Measurements were performed on cakes from each formulation (Table 1) the day after baking (after cooling and overnight holding). For testing, the crumb was sampled from the central region of each cake, and the crust was removed. The crumb was cut into uniform cubes (25 × 25 × 25 mm) (i.e., ~2.5 cm sided) to standardize geometry.

A two-cycle compression test (TPA) was applied with 50% target deformation (compression to 50% of the original cube height), trigger force 5 g, and 5 s recovery time between cycles. The pre-test, test, and post-test speeds were set to 1.0 mm/s. Hardness 1/2, adhesiveness, resilience, cohesiveness, springiness, gumminess, and chewiness were calculated from the force–time curve using established definitions [18].

The reported outputs were: Hardness 1 (g), the peak force during the first compression; Hardness 2 (g), the peak force during the second compression; Adhesiveness (mJ), the negative work associated with probe withdrawal (a measure of stickiness); Resilience, the ratio of energy recovered during the first decompression relative to the energy applied in the first compression; Cohesiveness, the ratio of the work of the second compression to that of the first (structural integrity under repeated deformation); Springiness (mm), the recovery distance between the end of the first compression and the start of the second; Gumminess (g), a derived parameter typically calculated as Hardness 1 × Cohesiveness; and Chewiness (mJ), a derived parameter typically calculated as Gumminess × Springiness (energy required to masticate a semi-solid food to a swallowable state) [18]. Force values are reported as grams-force (shown as g) as provided by the instrument/software (1 g = 0.00981 N).

#### 2.2.8. Sensory Analysis

Sensory evaluation was conducted using four formulations selected to represent the control and objective-relevant extremes of fat type/level and lipase–pre-emulsification conditions: L100–P0–Lip0, L100–P1–Lip100, L50–P1–Lip100, and S50–P1–Lip100 (see Table 1 for sample-code definitions). Cakes were prepared as described in the cake-making protocol and, consistent with the study workflow, were held until the following day prior to analyses.

The sensory panel comprised 15 trained panelists (age 25–69 years; 6 male, 9 female). Prior to participation, panelists were informed about the study procedures and provided written informed consent; participation was voluntary, with the right to withdraw at any time. Panelists reported no allergies to any ingredients used in the cake formulations, consistent with the allergen-risk briefing included in the participant information sheet. Evaluation was performed in a controlled session in which coded samples were tasted and/or smelled and scored on a 9-point hedonic scale (1 = “Disliked extremely” to 9 = “Liked extremely”) for the attributes taste, color, texture, and odor, with space provided for open comments [19].

Each coded sample was served as a standardized portion (~20 g; approximately one-quarter of a muffin) at room temperature on odor-free disposable plates. Between samples, palate cleansing and short breaks were provided (water and crackers), and panelists followed session rules intended to minimize sensory carryover (e.g., avoiding strongly flavored foods/beverages, smoking, gum, and perfume before the session). In addition to hedonic scoring, panelists provided an overall preference ranking by ordering the four coded samples from most to least preferred (ranking methodology aligned with ISO guidance) [20]. The testing environment and assessor training principles were aligned with ISO guidance for sensory test rooms and trained assessors [21]. Ethical considerations: The sensory evaluation involved only the tasting of coded cake samples in standard portion sizes by healthy adult volunteers. No interventions, biological sampling, or collection of health-related data were performed. Participants were fully informed about the procedures and potential allergen risks and provided written informed consent prior to participation. Participants could withdraw at any time without penalty. Individual responses were recorded using participant codes and analyzed in aggregated form; no identifiable personal information was included in the analytical dataset. Personal data handling followed the Turkish Personal Data Protection Law (KVKK, Law No. 6698) as described in the participant information sheet. As the study did not involve medicinal products, medical devices, or other clinical interventions, it falls outside the scope of the Turkish Regulation on Clinical Trials (Official Gazette, 13 April 2013, No. 28617).

#### 2.2.9. Statistical Analysis

All experiments were performed using two independent cake samples, with each measurement conducted at least three times to ensure reproducibility. Data are presented as mean values ± standard deviation (SD). Statistical analyses were carried out using JMP Pro 18 Student Version (SAS Institute Inc., Cary, NC, USA). A one-way analysis of variance (ANOVA) was employed to identify significant differences among formulations at a significance level of *p* < 0.05. When ANOVA indicated significant differences, Tukey’s Honestly Significant Difference (HSD) post hoc test was applied for pairwise comparisons. Interaction-like patterns discussed in the Results are based on descriptive comparisons and stratified visualization across factor-comparable formulation groups (e.g., matched pairs or within-group dose responses), rather than on a fitted factorial/DOE regression model.

## 3. Results

### 3.1. Moisture Content

**Figure 1:**
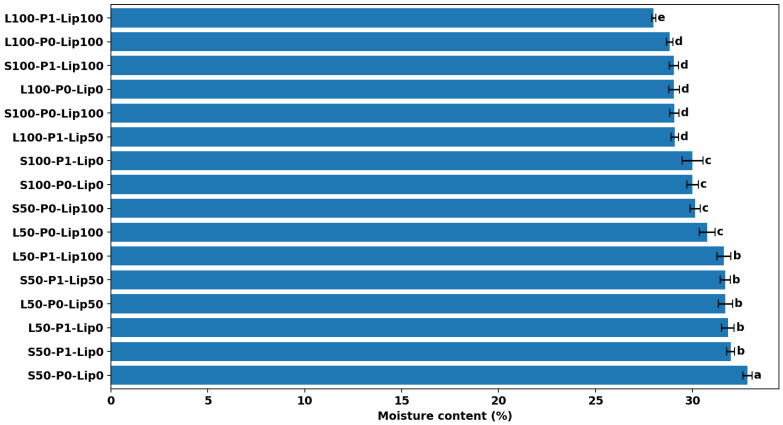
Moisture content (%) of cakes by formulation (mean ± SD). Different letters indicate significant differences among formulations (Tukey’s test, *p* < 0.05).

**Figure 2:**
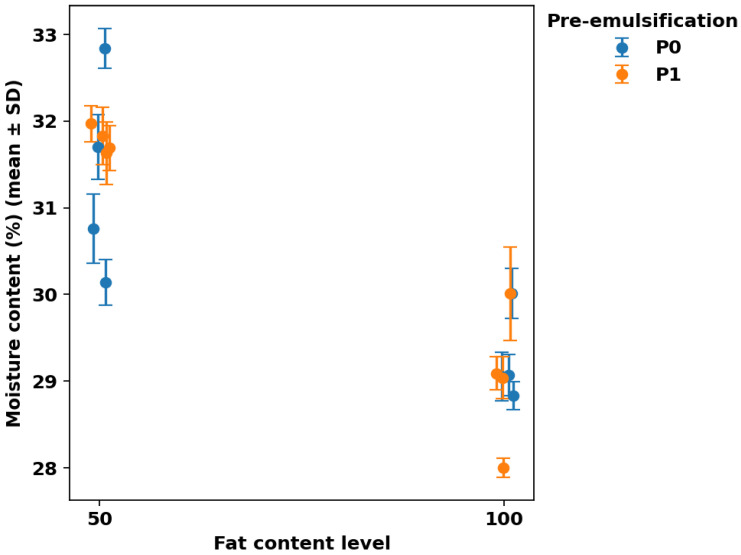
Main effect of fat level (50 vs. 100) and pre-emulsification (P0 vs. P1) on cake moisture content (%) (mean ± SD).

**Figure 3:**
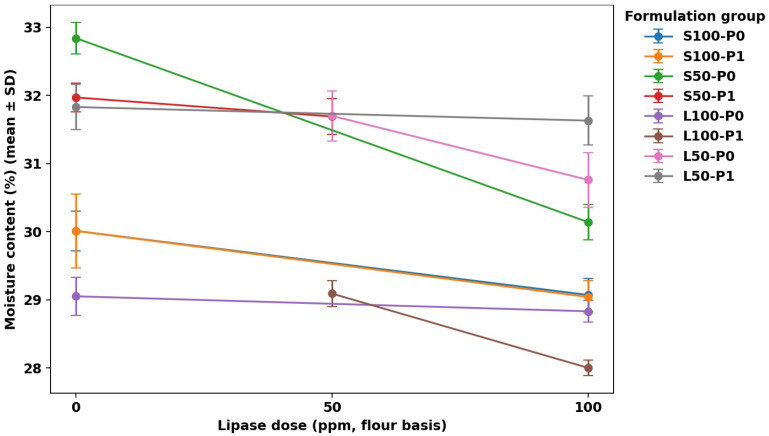
Moisture content (%) as a function of lipase dose (0, 50, 100 ppm; flour basis) within each formulation group (mean ± SD). Lines connect available design points only.

**Figure 4:**
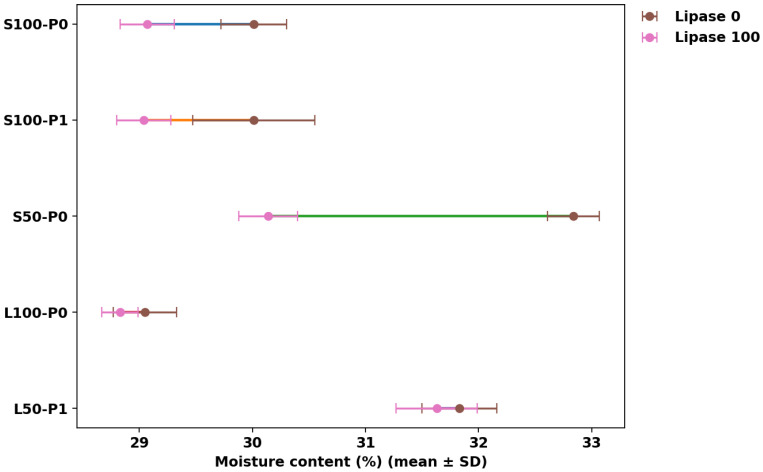
Within-group change in moisture content (%) from lipase 0 to 100 ppm for formulation groups containing both doses (mean ± SD). Solid colored segments connect the paired Lipase 0 and Lipase 100 means for each formulation to visualize the lipase-induced shift.

Moisture content results can be seen in Figure 1, Figure 2, Figure 3 and Figure 4. Figure 1 summarizes mean moisture content across all formulations with Tukey groupings, Figure 2 illustrates the main effects of fat level and pre-emulsification on moisture, Figure 3 shows the moisture–lipase dose response within each formulation group to visualize interactions, and Figure 4 isolates the within-group change in moisture from 0 to 100 ppm lipase where both doses are available.

Moisture content varied meaningfully across formulations (≈ 28.0–32.8%), indicating that the fat type/level and the applied processing route altered water retention during baking and/or the early post-bake equilibration period (Figure 1). This moisture window is comparable to values commonly reported for cake matrices produced under diverse formulation and process manipulations, including sponge cakes spanning ~28.5–36.6% moisture [22], sugar-reduced cakes reported at ~29.4–30.6% [23], 26.66–32.16% across formulation-driven quality optimization of cake crumb [24], and ~30.8% in control eggless cake systems [6]. The highest moisture was observed in the low-fat shortening system without pre-emulsification and lipase (S50-P0-Lip0: 32.84 ± 0.23%), whereas the lowest moisture occurred in the full-fat liquid system when pre-emulsification was combined with the highest lipase dose (L100-P1-Lip100: 28.00 ± 0.11%). The clustering of low-fat formulations toward higher moisture (Figure 2) is consistent with the central role of fat phase structuring and aeration in cake batter setting, where changes in fat functionality can shift water distribution and retention in the baked crumb [2,4].

Across most factor-comparable pairs, lipase addition reduced moisture, but the magnitude depended strongly on the fat type (Figure 3), confirming a fat×lipase interaction. The clearest within-group drop occurred in the highest-moisture baseline (S50-P0), where moisture decreased by ~2.7 percentage points from Lip0 to Lip100 (32.84 ± 0.23% to 30.14 ± 0.26%; *p* < 0.05), while the corresponding change was small or negligible in some full-fat liquid-oil conditions (e.g., L100-P0: 29.05 ± 0.28% to 28.83 ± 0.16%, not statistically significant). The consistent directionality of Lip0→Lip100 shifts (Figure 4) supports the interpretation that lipase-driven interfacial modification can promote structural changes that favor moisture loss (e.g., via more open, porous matrices and greater effective evaporation), a mechanism also reported when emulsifier-like interventions increase sponginess/volume yet lower moisture due to enhanced water loss during baking [25]. This aligns with work showing baking lipases act through lipid modification to alter batter/crumb functionality and can measurably affect baking water loss depending on formulation context [8], and with studies in low-fat cakes where lipase and emulsifiers modify structure and quality outcomes in a formulation-dependent manner [7,15].

Pre-emulsification effects were not uniform and depended on both fat type and lipase dose (fat×P and P×lipase interactions), as reflected by the divergence between groups in Figure 2 and Figure 3. In the sunflower-oil low-fat baseline, pre-emulsification lowered moisture at Lip0 (S50-P0-Lip0: 32.84 ± 0.23% vs. S50-P1-Lip0: 31.97 ± 0.21%), whereas at L50 with Lip100 it increased moisture relative to the non-pre-emulsified counterpart (L50-P1-Lip100: 31.63 ± 0.36% vs. L50-P0-Lip100: 30.76 ± 0.40%), suggesting that the impact of droplet pre-dispersion can either facilitate moisture loss (through aeration/porosity) or enhance water entrapment (through finer, more stable emulsified fat–aqueous structuring), depending on matrix composition. Comparable variability has been reported in emulsion-structured fat replacement systems, where initial moisture may be similar across formulations, but moisture retention/loss behavior can diverge with fat structuring strategy [26]. Because intermediate lipase doses and some P0/P1 baselines are not available for all fat types, inference on full dose–response and three-way interactions should remain conservative; nevertheless, the figures collectively indicate that lipase tends to reduce moisture most strongly in the high-moisture low-fat sunflower-oil condition (Figure 3 and Figure 4), while pre-emulsification modulates this direction and magnitude in a fat-system-specific manner (Figure 2 and Figure 3).

### 3.2. Color Measurement

**Figure 5:**
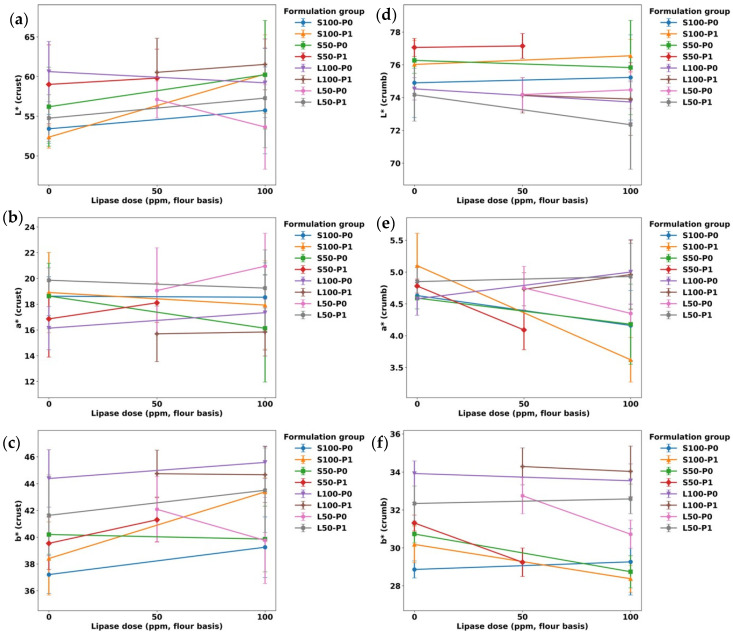
Changes in CIE Lab* color coordinates of cake crust (left column) and crumb (right column) as a function of lipase dose (ppm, flour basis) across formulation groups. Panels: (**a**) L* (crust), (**b**) a* (crust), (**c**) b* (crust); (**d**) L* (crumb), (**e**) a* (crumb), (**f**) b* (crumb).

**Figure 6:**
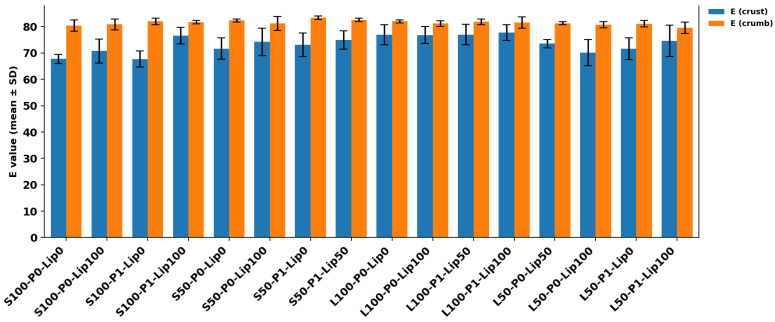
Crust and crumb color difference expressed as E (mean ± SD) for each formulation, reported separately for crust (blue) and crumb (orange).

Across all formulations, crust color was comparatively insensitive to the processing factors, whereas crumb color showed clearer formulation-dependent shifts (Table 2; Figure 5 and Figure 6). This divergence is expected because crust color development is dominated by surface dehydration and thermally driven browning (Maillard/caramelization) under baking conditions, while crumb color is additionally modulated by ingredient optics (fat droplet size/distribution, air cell structure, and light scattering) and internal moisture gradients [27]. Crust L* (52.36–61.53) and a* (15.70–20.95) did not separate statistically across treatments, indicating that neither lipase dose nor pre-emulsification measurably altered overall crust browning intensity in this dataset (Table 2). In contrast, crust b* (yellowness) was the parameter that most clearly differentiated formulations: L-coded 100-level samples clustered at the upper end (e.g., L100-P0-LIP100: 45.58 ± 1.21), while several S-coded controls were lower (e.g., S100-P0-LIP0: 37.21 ± 1.41). This pattern (also reflected by the relatively shallow slopes in Figure 5) suggests that the fat type/level is the dominant driver of crust yellowness, plausibly via lipid-phase color/optical contributions (including oil-associated yellowness and pigments) and fat-mediated light scattering, rather than via changes in browning chemistry [28,29].

Crust ∆E* remained statistically unchanged, reinforcing the practical inference that visual crust color was preserved across the factorial space (Figure 6; Table 2). This is aligned with emulsion-based fat-replacement work in pound cake where crust color often shows limited sensitivity even when crumb coordinates shift, likely because the crust overrides formulation subtleties through strong thermal gradients at the surface. Crumb L* showed a modest but meaningful spread (72.34–77.15). The lowest value occurred for L50-P1-LIP100: 72.34 ± 2.72, indicating a darker crumb relative to the highest-lightness group (S50-P1-LIP0: 77.06 ± 0.55). Because the crumb remains close to the boiling point while water is still present, conditions that limit Maillard-driven color formation, the observed darkening is more plausibly attributed to physical/optical effects such as changes in porosity/air-cell architecture and fat-phase dispersion that alter light scattering than to accelerated Maillard chemistry [27].

Crumb a* and b* captured clearer treatment signatures and interaction-like behavior. For example, S100-P1 exhibited a pronounced decrease in redness with lipase (a*: 5.10 ± 0.51 (a) → 3.62 ± 0.35 (c) from LIP0 to LIP100), whereas L100 samples remained comparatively stable around ~4.6–5.0. Meanwhile, crumb b* was consistently high in L100 formulations (~33–34) and lower in many S-coded samples (~28–31). This again points to fat type as a primary determinant of crumb yellowness, while lipase/pre-emulsification can modulate chroma within specific formulation contexts. These observations are consistent with fat-replacement literature in cakes: emulsion-mediated fat replacement can shift crumb L*, a*, and b* (often reducing chroma and/or changing lightness) through altered droplet/cell optics and water distribution, even when crust coordinates are largely unaffected [26].

**Figure 7:**
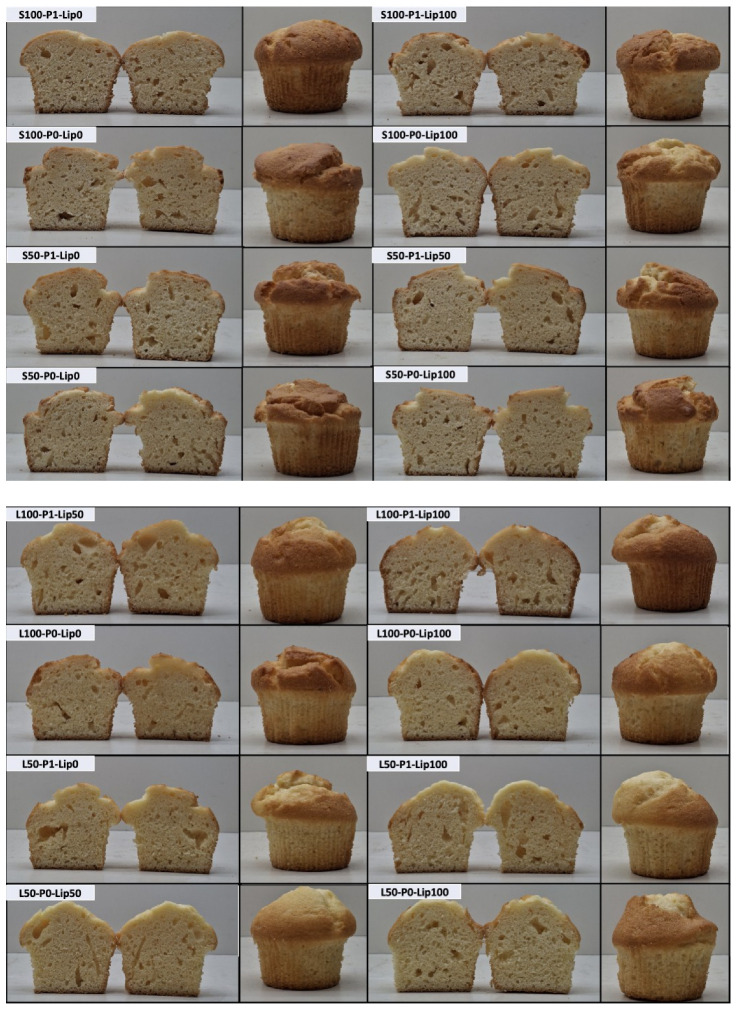
Visual analysis photographs of cake samples.

With respect to the study objective, the color results suggest that lipase addition and pre-emulsification can be used without systematically altering product appearance, although some formulation-specific crumb shifts do occur. At the crust level, appearance was stable across treatments: the lack of significant differences in crust L*, a*, and ∆E* indicates that neither lipase nor pre-emulsification measurably changed overall browning intensity, so the surface color cue perceived by consumers was essentially maintained. In contrast, the crumb responded more noticeably, but not in a uniform way. The clearest separations were observed in crumb chroma (a* and b*), and these differences tracked mainly with the fat type/level rather than with lipase or pre-emulsification alone. This implies that switching between shortening- and oil-based designs is likely to produce more visible crumb-color differences than adjusting processing aids within a given fat design (Figure 7).

L50-P1-Lip100 yielded the lowest crumb L*, suggesting that combining strong fat reduction with pre-emulsification and high lipase can reduce crumb lightness in this formulation space. This does not necessarily indicate greater internal browning; instead, it is more plausibly explained by optical changes driven by crumb structure. A denser crumb—lower void fraction and/or a finer gas-cell network—scatters less light and there-fore appears darker, and this effect can be strengthened if lipid droplets are more finely dispersed, further changing the internal refractive index contrasts [30,31]. These trends are consistent with the known role of baking lipases as batter improvers: by generating surface-active lipolysis products in situ, lipases partially reproduce emulsifier-like functionality at interfaces, altering how air cells and fat droplets are stabilized during mixing and baking. As a result, color changes in the crumb are expected to be context-dependent and to reflect formulation- and process-driven microstructural differences rather than a direct browning effect [8].

### 3.3. Volume and Visual Analysis

**Figure 8:**
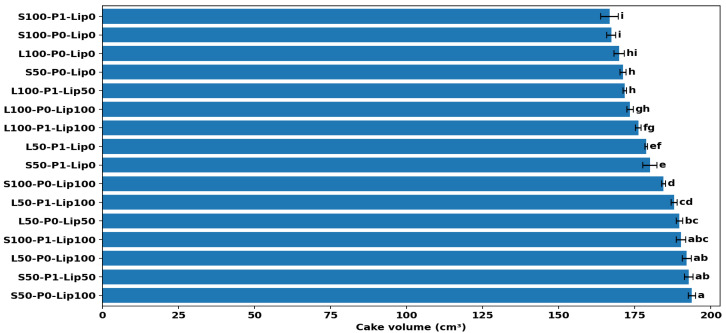
Cake volume (cm^3^) by formulation (mean ± SD) with Tukey group letters indicating significant differences among samples (*p* < 0.05).

**Figure 9:**
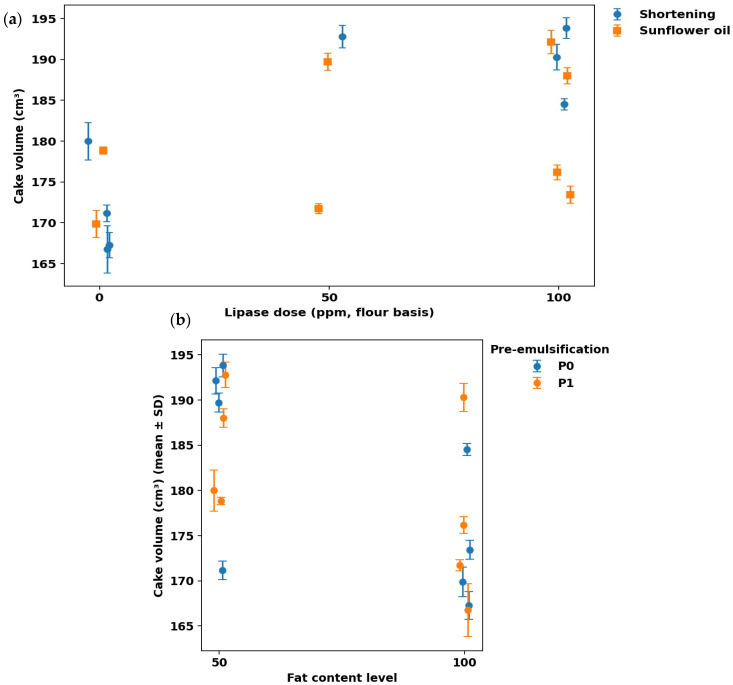
Cake volume responses summarized by factor effects: (**a**) volume (cm^3^) versus lipase dose (ppm, flour basis), shown separately for shortening- and sunflower oil–based systems (mean ± SD); (**b**) effect of fat content level and pre-emulsification (P0 vs. P1) on cake volume (cm^3^) across formulations (mean ± SD).

Lipase dose had the strongest overall effect on volume; however, the size and even the direction of this effect depended on the fat type/level and pre-emulsification condition (Figure 7, Figure 8 and Figure 9). Across all formulations, volumes ranged from ~167 to ~194 cm^3^. Across all formulations, cake volumes ranged from ~167 to ~194 cm^3^. The highest mean volume was obtained for S50–P0–Lip100, while the lowest mean volume was observed for S100–P1–Lip0 (Figure 8) corresponding to an increase of roughly. This ordering is directly relevant to the study objective because it shows that large expansion can be recovered (even maximized) within the reduced-fat region when lipase is used under an appropriate formulation context. The clearest treatment gain occurred in the shortening system, where Lip100 shifted volume from the lowest statistical group into the upper tier (S100-P1-Lip0: 166.75 ± 2.91 vs. S100-P1-Lip100: 190.28 ± 1.56), corresponding to an increase of roughly ~10–15% based on the measured mean values (Figure 8). This pattern is consistent with previous low-fat cake studies reporting that lipase-based improvers can increase expansion but do so in a formulation-dependent manner [8,15]. The photographs provide a convergent, product-level confirmation of these ranking shifts: low-volume shortening controls show a more compact crumb section and a flatter overall profile, whereas the Lip100 counterpart appears more expanded and aerated (Figure 7).

The response in sunflower-oil systems was more constrained, and this contrast clarifies why lipase cannot be treated as a uniform volume booster (Figure 8 and Figure 9). Even at Lip100, L100 remained comparatively low, indicating that the benefit of lipase depends on fat phase and fat level rather than scaling linearly with dose. This behavior aligns with mechanistic work showing that lipase outcomes follow how lipolysis products redistribute across the batter’s interfacial network, which is inherently shaped by the available substrates and the architecture of the dispersed phases [10,11]. The image set supports the same direction qualitatively, with L100 samples showing smaller apparent expansion than the highest-volume low-fat shortening cases (Figure 7).

Pre-emulsification further altered the lipase response, with interaction-like patterns evident in formulation-level comparisons (Figure 8) and summarized as factor-level trends (Figure 9). In low-fat shortening without lipase, pre-emulsification increased volume (S50-P0-Lip0: 171.18 ± 1.02 vs. S50-P1-Lip0: 180.00 ± 2.29), which is consistent with improved dispersion and air incorporation when the structural contribution of fat is reduced; visually, the S50-P1-Lip0 crumb sections appear more expanded than the corresponding P0 condition (Figure 7). This direction matches the established role of interfacial aids in stabilizing entrained air during mixing and early baking, particularly when the fat phase is less able to provide shortening-like aeration functionality [32]. However, the same processing step did not improve volume universally. In low-fat sunflower oil at high lipase dose, pre-emulsification reduced volume (L50-P0-Lip100: 192.12 ± 1.44; ab vs. L50-P1-Lip100: 188.00 ± 1.00; cd), indicating that increasing interfacial area can become counterproductive once the lipid phase is already strongly modified by enzymatic action; the paired photographs show a modest reduction in apparent expansion with P1 that is consistent with the direction of the volumetric shift (Figure 7). This is consistent with earlier low-fat cake work where lipase–emulsifier performance depended on the specific fat/reducer matrix and processing route [15].

A coherent mechanistic explanation emerges when the numerical trends are considered together with the formulation dependence. Lipase likely improved gas-cell stability by generating surface-active lipolysis products in situ, thereby strengthening the foam–emulsion network during expansion. Lipidomic evidence from cake batters supports that baking lipases reshape the emulsifying lipid pool (including increases in monoacylglycerol-type species), which can translate into improved structure setting and product quality, but only when substrate availability and formulation constraints permit effective interfacial reorganization [10,11]. The contrasting behavior between shortening and liquid oil is consistent with the broader principle that liquid oils often require stronger interfacial assistance to emulate shortening-like functionality; therefore, the largest gains appear in conditions where lipase and/or pre-emulsification plausibly compensate for reduced structural contributions of the fat phase (Figure 7, Figure 8 and Figure 9), matching reports that lipase/emulsifier effects in low-fat cakes are strongly context-specific [8,15]. These mechanistic interpretations are based on the observed quality outcomes and established lipase functionality; lipolysis was not quantified directly in the batter (e.g., by acid value or lipid profiling).

### 3.4. Texture Profile Analysis

**Figure 10:**
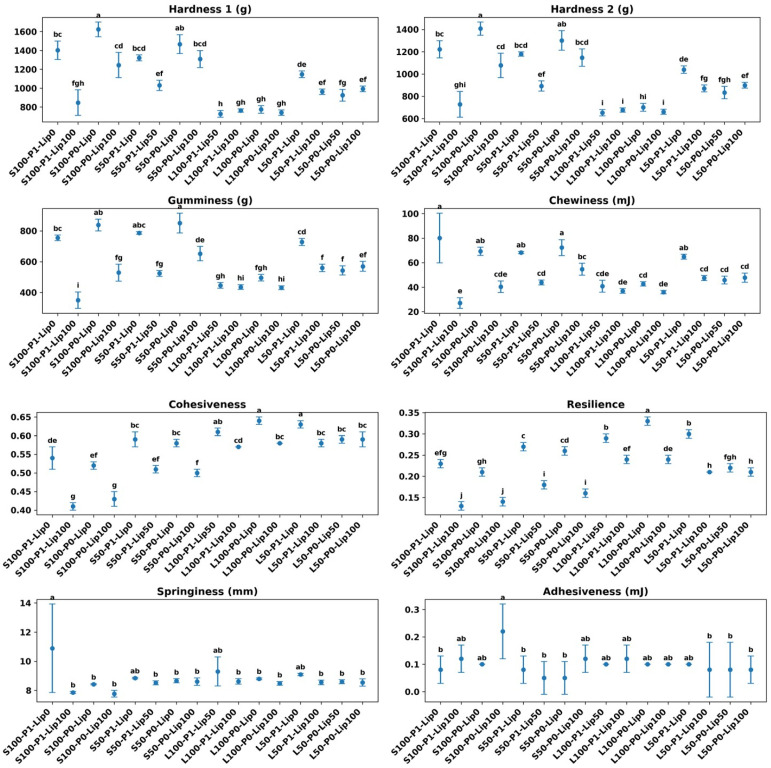
Texture profile analysis (TPA) of cakes: Hardness 1/2, gumminess, chewiness, cohesiveness, resilience, springiness, and adhesiveness (mean ± SD). Different letters indicate Tukey groups (*p* < 0.05).

**Figure 11:**
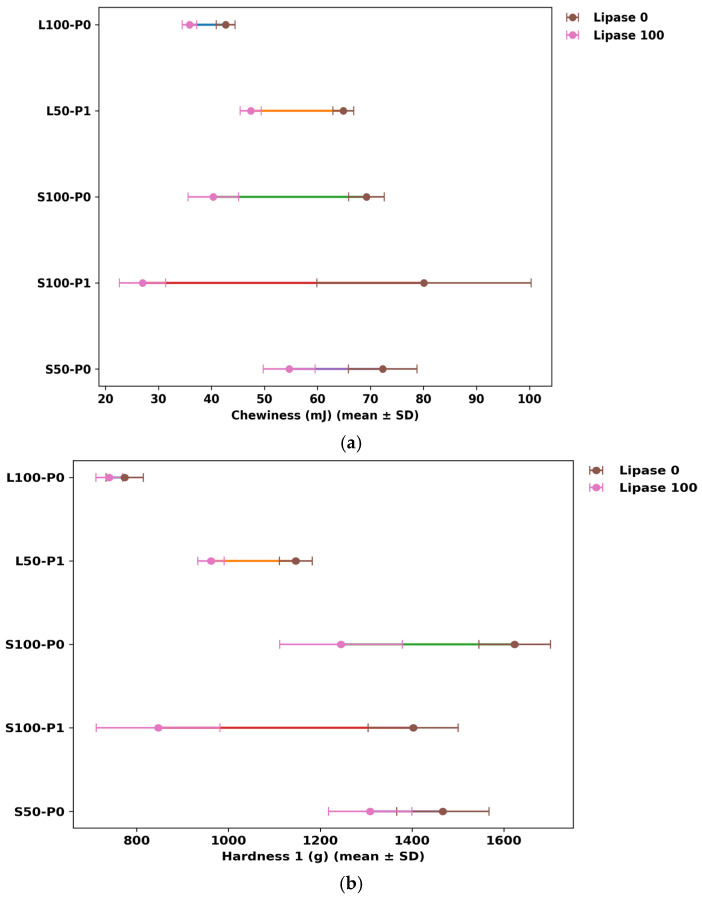
Lipase effect within matched formulations (Lip0 → Lip100) on (**a**) chewiness (mJ) and (**b**) hardness 1 (g). Points are means ± SD for each formulation group (fat type/level × pre-emulsification) measured at Lip0 and Lip100. Solid colored segments connect the paired Lipase 0 and Lipase 100 means for each formulation to visualize the lipase-induced shift.

**Figure 12:**
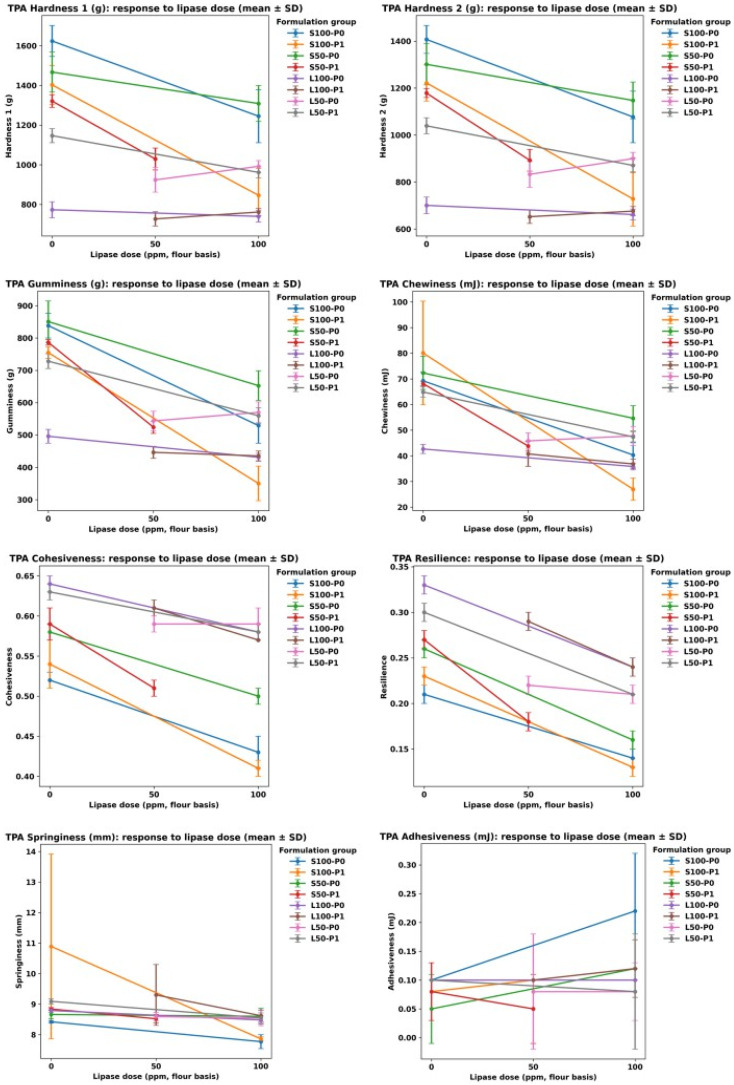
Response of texture profile analysis (TPA) parameters to lipase dose (0–100 ppm, flour basis) across formulation groups (fat type/level × pre-emulsification): hardness 1 (g), hardness 2 (g), gumminess (g), chewiness (mJ), cohesiveness, resilience, springiness (mm), and adhesiveness (mJ). Points are means ± SD; lines connect available design points within each formulation group.

Across formulations, texture was dominated by fat type and then modulated by lipase dose and pre-emulsification (Figure 10, Figure 11 and Figure 12). Shortening-based cakes exhibited substantially higher hardness and mastication demand than sunflower-oil systems, consistent with the well-known role of plastic fats in structuring batter aeration and setting the crumb matrix [2,4]. This contrast is clear in the extremes: the firmest crumb occurred in S100-P0-Lip0 (Hardness 1 = 1623.75 ± 77.93 g), whereas the softest shortening condition was achieved after lipase in the pre-emulsified full-fat sample (S100-P1-Lip100; Hardness 1 = 846.50 ± 135.06 g), indicating a shift from a highly resistant crumb to a markedly more tender structure under otherwise comparable fat level (Figure 10, Figure 11 and Figure 12). Such fat-system dependence aligns with prior cake studies showing that lipid physical state and interfacial functionality govern gas-cell stabilization during mixing and early baking, thereby controlling crumb mechanical response [2,32,33].

Lipase consistently reduced hardness-related parameters in shortening systems, with the clearest effects at Lip100 and stronger responses when pre-emulsification was applied. Within S100, increasing lipase from 0 to 100 ppm lowered not only Hardness by 50% but also gumminess and chewiness, with the most pronounced softening occurring in P1 compared with P0, suggesting a meaningful P×lipase interaction. Mechanistically, this pattern is consistent with lipase-driven generation of surface-active partial glycerides and free fatty acids that enhance emulsification and gas-cell stabilization, promoting a more expanded, less mechanically resistant crumb—effects repeatedly reported for lipases used as “clean-label” emulsifier alternatives in cakes [7,8,15]. In low-fat shortening (S50), lipase still lowered mastication demand, but the magnitude depended on the available design points (not all groups include all doses), indicating that the lipase response is not purely additive and likely depends on the initial interfacial and aeration state created by fat reduction and processing history (Figure 12) [2,12,15].

The same interventions that softened the crumb also introduced a structural-recovery trade-off that is highly relevant to “quality preservation” in low-fat design. In shortening systems, lipase lowered cohesiveness and resilience alongside hardness (Figure 10 and Figure 12), implying a crumb that is easier to compress but less able to recover and maintain internal integrity under deformation. This is particularly evident in the pre-emulsified full-fat shortening condition, where cohesiveness dropped from 0.54 ± 0.03 at Lip0 to 0.41 ± 0.01 at Lip100, and resilience decreased from 0.23 ± 0.01 to 0.13 ± 0.01. In practical terms, the product becomes more tender but potentially more prone to crumbliness or weaker elastic response during handling and mastication—an outcome consistent with the concept that excessive interfacial softening (via strong surfactant effects) can produce thinner lamellae around gas cells and a less continuous load-bearing network once starch gelatinization and protein aggregation set the crumb [2,33,34]. Emulsifier-oriented cake work similarly notes that changes improving aeration/tenderness can simultaneously alter cohesiveness and resilience by shifting how the protein–starch matrix consolidates around stabilized bubbles [15,32].

Sunflower-oil systems (L100/L50) displayed a different balance: markedly lower hardness and gumminess overall, while maintaining comparatively high cohesiveness and resilience. This combination is compatible with prior observations that replacing plastic fats with liquid oils alters crumb mechanics not only via aeration differences but also via lubrication and lipid–matrix interactions, often yielding softer bite while changing deformation recovery in formulation-dependent ways [4,35]. Importantly, lipase effects on L100 were small relative to shortening (Figure 10), consistent with a ceiling effect when a liquid oil system already provides high interfacial mobility and internal lubrication; additional enzymatically generated surfactants may then deliver limited incremental change in measured hardness, while still modulating network recovery modestly [8,33]. Pre-emulsification likewise exerted smaller shifts in the oil systems than in shortening, supporting the interpretation that the key leverage of P1 is strongest when it restructures a semi-solid fat phase into a finer, more effective aerating/emulsifying dispersion [2,32].

Stickiness-related behavior was generally low across conditions, but the occasional rise in adhesiveness at high lipase in shortening signals another formulation-specific trade-off that may matter for consumer perception. A plausible explanation is that strong lipase activity increases the pool of mobile surface-active lipids, which can promote perceived tackiness during compression even while lowering hardness—an effect reported in lipase-based cake improvement studies where crumb/batter rheology and sur-face properties shift in parallel [7,10]. Springiness was comparatively stable around a narrow band in most formulations, implying that the primary changes in “eating effort” were driven more by hardness/gumminess and by reduced structural recovery than by rebound distance per se, consistent with how TPA components are typically governed by the balance between starch gel strength and protein network continuity rather than by single-factor changes alone [2,34].

Overall, the data indicate that lipase is an effective lever to improve low-fat cake texture by lowering hardness and mastication demand, and that pre-emulsification can amplify this benefit in shortening systems. The key constraint is that the strongest softening coincides with reduced cohesiveness and resilience, implying that optimization should target a tender-yet-cohesive window rather than maximal softening. Because not every fat×P combination contains the full lipase dose set, interaction conclusions are strongest where matched Lip0/Lip100 comparisons exist; nonetheless, the consistency of the directionality across multiple TPA endpoints supports a coherent mechanism centered on interfacial modification and altered crumb consolidation, in agreement with established lipase/emulsifier functionality in cake systems [7,8,15,33].

### 3.5. Sensory Analysis

**Figure 13:**
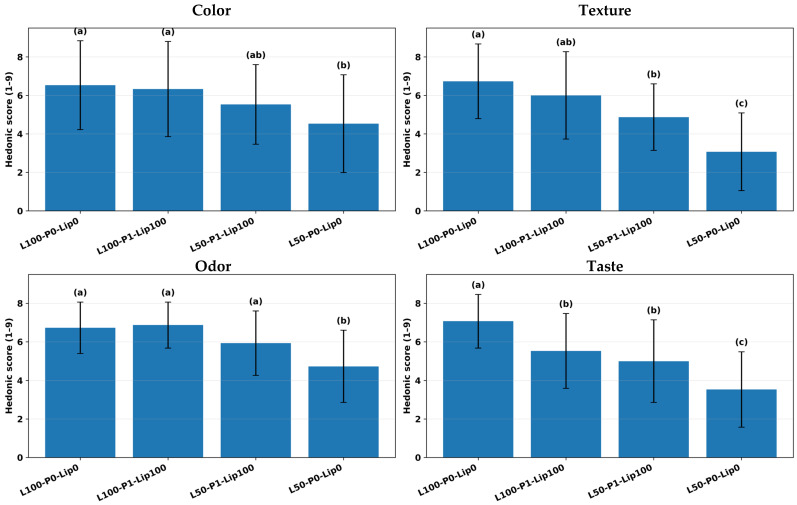
Hedonic sensory scores (mean ± SD) for color, texture, aroma, and taste (9-point scale) of selected formulations; different letters indicate significant differences among formulations within each attribute (Tukey, *p* < 0.05).

Reducing fat without additional structuring produced a clear sensory penalty, consistent with the central role of fat in aeration, tenderness, lubrication, and flavor delivery in batter-type cakes. The low-fat control (L50-P0-Lip0) clustered at the bottom across attributes, with especially depressed texture and taste (3.07 ± 2.02, and 3.53 ± 1.96), whereas the full-fat reference (L100-P0-Lip0) remained highest (texture 6.73 ± 1.94, taste 7.07 ± 1.39) (Figure 13). This magnitude of drop aligns with consumer work on reduced-fat cakes showing that fat reduction disproportionately lowers liking of texture and overall acceptance unless compensatory structuring (e.g., emulsifier-enabled stabilization) is introduced [2].

**Figure 14:**
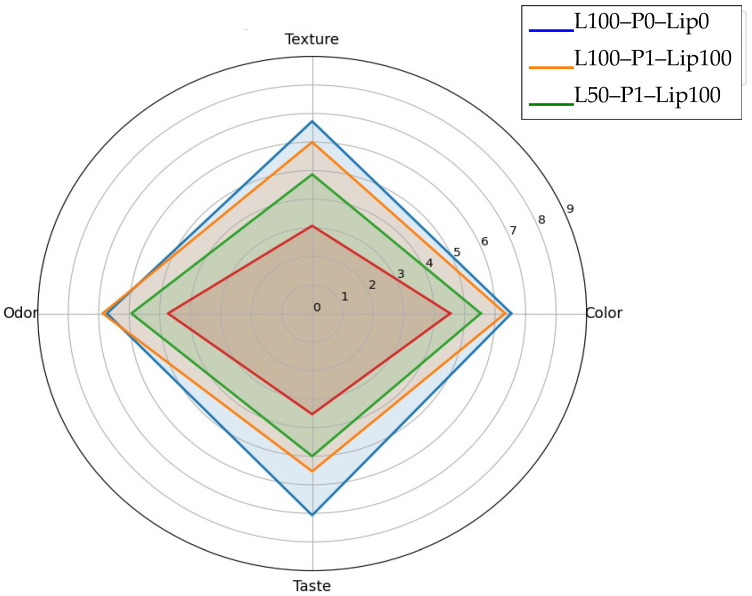
Radar chart of the sensory profile (mean hedonic scores on a 9-point scale) for color, texture, odor, and taste across selected formulations.

**Figure 15:**
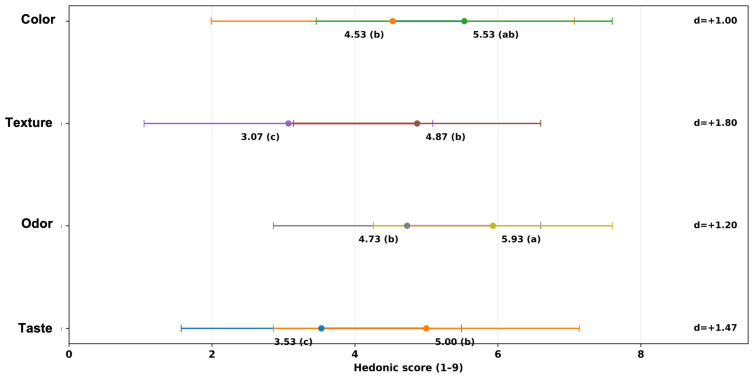
Paired comparison of low-fat improvement (L50-P0-Lip0 → L50-P1-Lip100) showing mean ± SD hedonic scores (9-point scale) with Tukey group letters for each attribute; Cohen’s *d* is reported as the standardized effect size for each attribute.

Against that baseline, the combined strategy of pre-emulsification with lipase at low fat substantially shifted acceptance upward, supporting the study’s main objective. Moving from L50-P0-Lip0 to L50-P1-Lip100 increased color and aroma into higher groupings and, critically, lifted texture from 3.07 ± 2.02 to 4.87 ± 1.73 and taste from 3.53 ± 1.96 to 5.00 ± 2.14 (Figure 13). The sensory fingerprint visualization highlights that this improvement was broad rather than attribute-specific, with the low-fat formulation’s profile shifting away from uniformly low z-scores toward the full-fat pattern (Figure 14), while the paired contrast plot indicates a large practical gain in texture and taste (∆ ≈ +1.8 and +1.5 hedonic units, respectively) (Figure 15). Mechanistically, this pattern is consistent with lipase-driven formation of more surface-active lipid species that can partially substitute for conventional emulsifiers at interfaces, improving dispersion and structural uniformity, while pre-emulsification can further support droplet-size reduction and more homogeneous fat distribution—both changes that are expected to translate into improved mouthfeel and more efficient flavor release in reduced-fat matrices [15].

The same treatment also underscored formulation dependence and a potential trade-off at higher fat: although L100-P1-Lip100 maintained high color and aroma (6.33 ± 2.47 and 6.87 ± 1.19), taste decreased from 7.07 ± 1.39 in L100-P0-Lip0 to 5.53 ± 1.94 (Figure 13), indicating that “more structuring chemistry” is not universally beneficial. Prior work notes that lipase performance depends on formulation and enzyme specificity and that excessive or mismatched lipolysis can impair sensory quality via altered lipid-derived flavor balance, even when physical quality improves [8]. The available sensory set is intentionally sparse (four formulations), so the relative contributions of pre-emulsification versus lipase cannot be cleanly separated here; nevertheless, the direction and size of the low-fat shift (Figure 13, Figure 14 and Figure 15) strongly support the conclusion that the combined approach is an effective route to recover low-fat consumer-relevant attributes, while also motivating dose/formulation optimization to avoid taste suppression under full-fat conditions [5].

## 4. Conclusions

In this study, an interfacial design strategy was evaluated to preserve cake quality under fat reduction and/or substitution of shortening with sunflower oil. The study demonstrated that lipase is a powerful functional lever in reduced-fat cake systems and that its performance is strongly formulation- and process-dependent.

Across the tested conditions, lipase-driven in situ generation of surface-active lipid species consistently improved structure-related quality, most clearly by reducing hardness-related texture parameters and lowering mastication demand, while supporting expansion and crumb refinement in selected matrices. The benefits were most pronounced when the baseline interfacial stability was constrained (e.g., reduced-fat and/or shortening-based systems), consistent with the proposed mechanism that lipase compensates for diminished shortening functionality by strengthening the foam–emulsion network through interfacial modification. Results suggest that pre-emulsification modulated the response to lipase in a formulation-dependent manner by increasing accessible interfacial area and improving fat dispersion prior to mixing, but its effect was not universally positive. The observed interactions indicate that optimal outcomes require matching the process route (pre-emulsification vs. direct addition) to the fat type and fat level; in some cases, excessive interfacial activity may introduce trade-offs such as reduced cohesiveness/resilience or sensory penalties at high-fat/high-lipase conditions.

Overall, the results support the central conclusion that pre-emulsification-enabled lipase action offers an effective route to recover key consumer-relevant attributes in low-fat cakes without relying on conventional emulsifiers. Future work should focus on dose optimization within each fat type, direct characterization of batter microstructure and interfacial composition, and extended storage studies to quantify staling kinetics and to define a tender-yet-cohesive quality window that maximizes acceptance.

## Figures and Tables

**Table 1:** Experimental design and sample coding for cake formulations.

Sample Code	Fat Type	Fat Level	Pre-Emulsification	Lipase (Ppm; Flour Basis)
S100–P0–Lip0	Shortening	Normal	No	0
	(solid)			
S100–P0–Lip100	Shortening	Normal	No	100
	(solid)			
S100–P1–Lip0	Shortening	Normal	Yes	0
	(solid)			
S100–P1–Lip100	Shortening	Normal	Yes	100
	(solid)			
S50–P0–Lip0	Shortening	−50%	No	0
	(solid)			
S50–P0–Lip100	Shortening	−50%	No	100
	(solid)			
S50–P1–Lip0	Shortening	−50%	Yes	0
	(solid)			
S50–P1–Lip50	Shortening	−50%	Yes	50
	(solid)			
L100–P0–	Sunflower oil	Normal	No	0
Lip0 (*control*)	(liquid)			
L100–P0–Lip100	Sunflower oil	Normal	No	100
	(liquid)			
L100–P1–Lip50	Sunflower oil	Normal	Yes	50
	(liquid)			
L100–P1–Lip100	Sunflower oil	Normal	Yes	100
	(liquid)			
L50–P0–Lip50	Sunflower oil	−50%	No	50
	(liquid)			
L50–P0–Lip100	Sunflower oil	−50%	No	100
	(liquid)			
L50–P1–Lip0	Sunflower oil	−50%	Yes	0
	(liquid)			
L50–P1–Lip100	Sunflower oil	−50%	Yes	100
	(liquid)			

**Table 2:** Crust and crumb color parameters (CIE Lab* and ∆E*) of cakes by formulation (mean ± SD). Different lowercase letters within the same column indicate significant differences among formulations (Tukey’s HSD, *p* < 0.05).

	CRUST				CRUMB			
Sample	L*	a*	b*	E*	L*	a*	b*	E*
S100-P1-	52.36 ±	18.91 ±	38.41 ±	67.73 ±	76.02 ±	5.10 ±	30.18 ±	80.40 ±
Lip0	1.40 a	3.12 a	2.73 cd	1.75 a	1.47 ab	0.51 a	0.96 defg	2.09 ab
S100-P1-	60.31 ±	17.93 ±	43.38 ±	70.75 ±	76.55 ±	3.62 ±	28.37 ±	80.86 ±
Lip100	4.95 a	3.43 a	1.06 abc	4.57 a	1.00 ab	0.35 c	0.71 g	2.07 ab
S100-P0-	53.41 ±	18.62 ±	37.21 ±	67.69 ±	74.90 ±	4.63 ±	28.86 ±	81.96 ±
Lip0	1.81 a	1.51 a	1.41 d	3.05 a	2.11 ab	0.21 ab	0.45 fg	1.18 ab
S100-P0-	55.73 ±	18.54 ±	39.25 ±	76.56 ±	75.23 ±	4.16 ±	29.26 ±	81.72 ±
Lip100	5.48 a	2.67 a	2.26 bcd	3.08 a	2.60 ab	0.55 abc	1.75 efg	0.69 ab
S50-P1-	59.01 ±	16.86 ±	39.54 ±	71.65 ±	77.06 ±	4.78 ±	31.31 ±	82.35 ±
Lip0	4.98 a	2.97 a	1.95 bcd	4.04 a	0.55 a	0.11 ab	0.42 bcde	0.57 ab
S50-P1-	59.80 ±	18.11 ±	41.30 ±	74.23 ±	77.15 ±	4.09 ±	29.24 ±	81.21 ±
Lip50	3.66 a	1.53 a	1.64 abcd	5.19 a	0.76 a	0.31 bc	0.75 efg	2.63 ab
S50-P0-	56.19 ±	18.63 ±	40.20 ±	73.10 ±	76.27 ±	4.59 ±	30.73 ±	83.32 ±
Lip0	4.98 a	2.54 a	1.47 abcd	4.41 a	0.79 ab	0.17 abc	0.47 cdef	0.63 a
S50-P0-	60.23 ±	16.13 ±	39.86 ±	74.93 ±	75.83 ±	4.18 ±	28.74 ±	82.61 ±
Lip100	6.85 a	4.18 a	2.45 bcd	3.43 a	2.88 ab	0.63 abc	0.85 fg	0.67 ab
L100-P1-	60.53 ±	15.70 ±	44.74 ±	76.88 ±	74.14 ±	4.73 ±	34.29 ±	82.02 ±
Lip50	4.30 a	2.16 a	1.74 ab	3.84 a	1.08 ab	0.26 ab	0.97 a	0.57 ab
L100-P1-	61.53 ±	15.84 ±	44.66 ±	76.80 ±	73.91 ±	4.96 ±	34.03 ±	81.16 ±
Lip100	3.20 a	1.87 a	2.09 ab	3.27 a	2.22 ab	0.54 ab	1.33 a	1.10 ab
L100-P0-	60.62 ±	16.14 ±	44.38 ±	76.95 ±	74.53 ±	4.58 ±	33.92 ±	81.82 ±
Lip0	3.81 a	1.67 a	2.14 ab	3.88 a	0.68 ab	0.26 abc	0.66 a	1.07 ab
L100-P0-	59.21 ±	17.34 ±	45.58 ±	77.71 ±	73.73 ±	5.00 ±	33.54 ±	81.53 ±
Lip100	4.39 a	2.91 a	1.21 a	3.00 a	1.24 ab	0.51 ab	0.89 ab	2.16 ab
L50-P1-	54.75 ±	19.86 ±	41.63 ±	73.55 ±	74.17 ±	4.85 ±	32.34 ±	81.23 ±
Lip0	2.95 a	0.96 a	3.01 abcd	1.60 a	1.61 ab	0.25 ab	0.92 abcd	0.57 ab
L50-P1-	57.28 ±	19.25 ±	43.50 ±	70.08 ±	72.34 ±	4.93 ±	32.58 ±	80.67 ±
Lip100	6.26 a	2.97 a	3.14 abc	4.96 a	2.72 b	0.52 ab	0.78 abc	1.14 ab
L50-P0-	57.10 ±	19.06 ±	42.08 ±	71.60 ±	74.18 ±	4.75 ±	32.75 ±	81.07 ±
Lip50	2.33 a	3.32 a	2.45 abcd	4.20 a	1.03 ab	0.34 ab	0.95 abc	1.25 ab
L50-P0-	53.63 ±	20.95 ±	39.76 ±	74.57 ±	74.47 ±	4.35 ±	30.72 ±	79.51 ±
Lip100	5.33 a	2.56 a	3.22 bcd	5.93 a	1.14 ab	0.15 abc	0.74 cdef	2.18 b

## Data Availability

The original contributions presented in the study are included in the article, further inquiries can be directed to the corresponding author.
